# Pancreas Divisum With Santorinicele: A Rare Cause of Recurrent Acute Pancreatitis

**DOI:** 10.7759/cureus.82498

**Published:** 2025-04-18

**Authors:** Fatimah A Alkhamis, Mohammed T Al Ali, Ahmet Aslan

**Affiliations:** 1 Radiology, King Fahad General Hospital, Al Hofuf, SAU; 2 Radiology, King Hamad University Hospital, Muharraq, BHR

**Keywords:** magnetic resonance cholangiopancreatography, magnetic resonance imaging, pancreatic divisum, pancreatitis, santorinicele

## Abstract

Santorinicele is a cystic dilatation at the terminal end of the pancreatic duct of Santorini, which is a rare condition mostly associated with pancreatic divisum. Patients with Santorinicele may present with recurrent pancreatitis due to elevated intraductal pressure and outflow obstruction of pancreatic secretions. Different imaging modalities, including magnetic resonance imaging (MRI) and magnetic resonance cholangiopancreatography (MRCP), are useful tools in identifying the causes of recurrent pancreatitis, and other pancreatic pathology and anatomical variants. Here, we present a case of recurrent acute pancreatitis caused by pancreas divisum with Santorinicele and discuss the role of medical imaging in the diagnosis.

## Introduction

Santorinicele was first described in 1994 by Eisen et al. [1] as a cystic dilatation of the terminal end of the dorsal duct of the pancreas, also called the duct of Santorini, located immediately proximal to the minor papilla. The nomenclature is analogous to ureterocele and choledochocele in their respective locations. It is a rare condition and a recognized cause of recurrent pancreatitis attacks [2,3]. It is mostly associated with pancreas divisum, even though a few cases of Santorinicele without pancreas divisum have been reported [4,5]. Different imaging modalities, including magnetic resonance imaging (MRI) and magnetic resonance cholangiopancreatography (MRCP), are useful in identifying the causes of recurrent pancreatitis, and pancreatic ductal anatomy and any anatomical variant or pathology [5-7]. In this case report, we present a case of recurrent acute pancreatitis caused by pancreas divisum with Santorinicele and discuss the role of medical imaging in the diagnosis.

## Case presentation

A 77-year-old female patient with medical comorbidities including type 2 diabetes mellitus, dyslipidemia, hypertension, ischemic heart disease, who had a history of acute pancreatitis, presented to the emergency department in December 2021 complaining of epigastric abdominal pain of acute onset, which was continuous, radiating to the flanks and associated with vomiting and loose motions. Upon examination, the patient was afebrile with no jaundice. The abdominal examination revealed tenderness in the epigastric region. Laboratory workup showed elevated serum lipase level (6832.1 U/L), which led to the diagnosis of acute pancreatitis.

**Figure 1:**
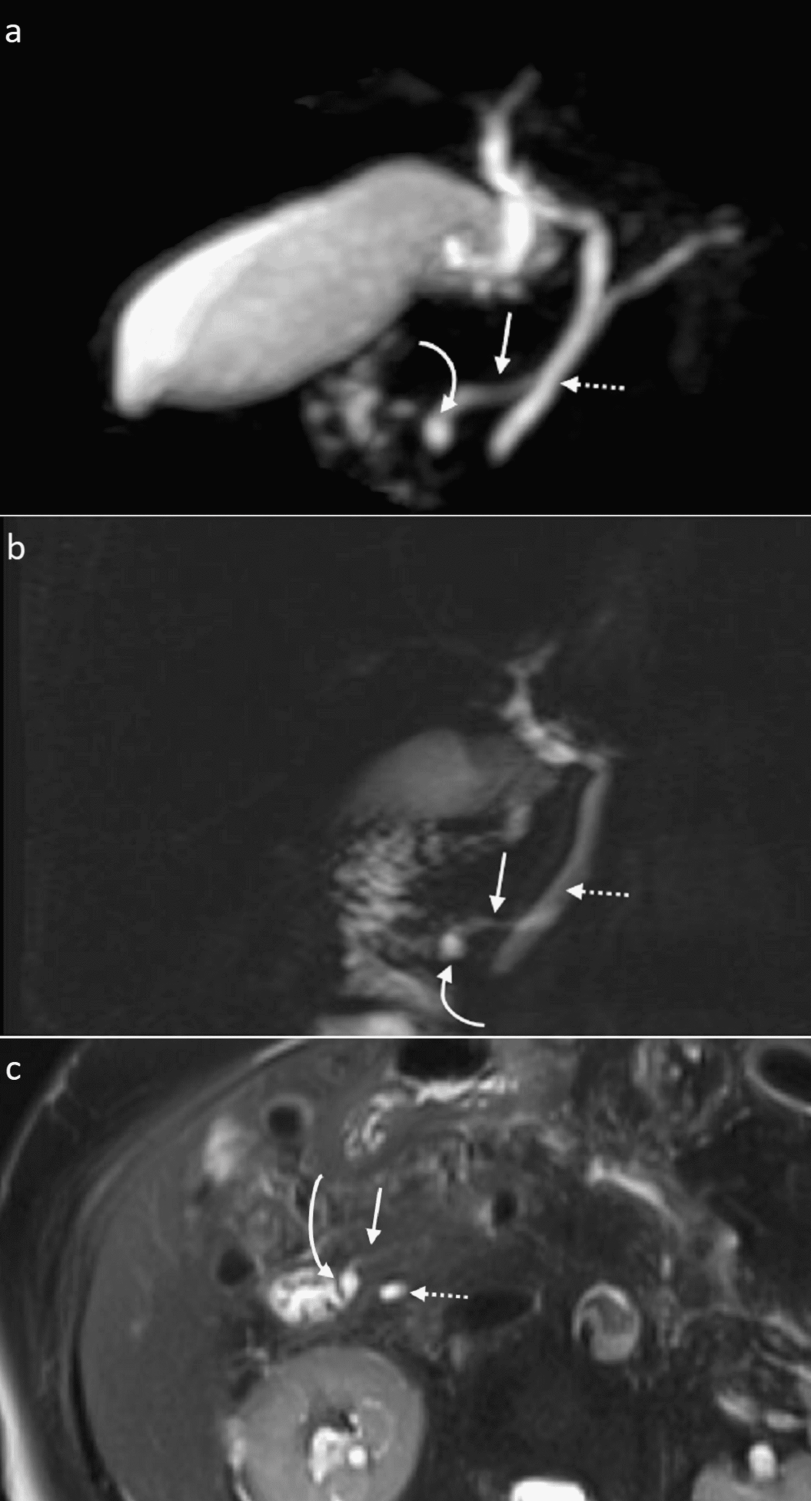
MRCP thick slap (a, b) and T2-weighted fat-saturated axial view (c) show focal dilatation indicative of Santorinicele (curved arrow) at the terminal end of the dorsal duct (straight arrow), which drains into the minor papilla. The common bile duct (dotted arrow) is seen draining separately into the major papilla. MRCP: magnetic resonance cholangiopancreatography

In view of the patient’s history of recurrent pancreatitis and lack of precipitating risk factors such as alcohol consumption or history of cholelithiasis, MRI of the pancreas and MRCP were performed to look for any structural anomaly or obstructive causes. MRCP images confirmed the presence of a pancreatic duct anatomical variant in which pancreas divisum with Santorinicele was demonstrated. The common bile duct is shown draining into the major papilla, whereas the pancreatic dorsal duct (duct of Santorini) is seen draining separately and proximally into the minor papilla. The Santorinicele is seen as a focal saccular dilation at the dorsal duct’s terminal end (Figure 1).

**Figure 2:**
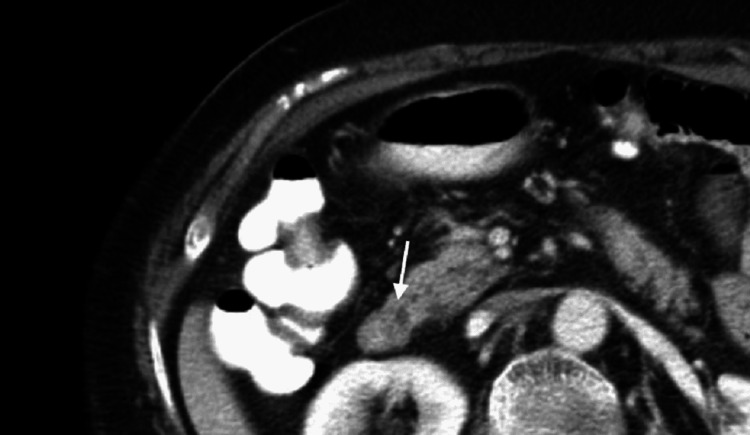
CT abdomen axial view in the venous phase shows a hypodense focal dilatation at the termination of the dorsal pancreatic duct, consistent with a Santorinicele (arrow).

Remarkably, the Santorinicele was even detectable on CT scan images that the patient underwent in 2014 after experiencing a pancreatitis attack when assessed retrospectively (Figure 2). Eventually, the patient was discharged home after having satisfactory symptomatic improvement with conservative management. A plan for endoscopic retrograde cholangiopancreatography (ERCP) with papillary sphincterotomy was considered if the patient develops a further acute pancreatitis attack. The patient hasn't had any further attacks of pancreatitis and is currently doing well. Therefore, no intervention has been needed.

## Discussion

Pancreatitis is a frequently encountered disease that significantly impacts patients’ quality of life. It has different etiological factors, with heavy alcohol consumption and cholelithiasis being the most common factors [8,9].

Pancreas divisum with Santorinicele has been suggested as a rare cause of recurrent acute pancreatitis [2,4,10]. Pancreas divisum is the most common pancreatic ductal variation [11-13] and is strongly associated with Santorinicele, occurring in 77.1% of affected patients [14].

Santorinicele is more commonly found in older age groups, with a reported mean age of 73.6 years (range: 67-85 years) in patients without pancreas divisum and 69.2 years (range: 56-78 years) in patients with pancreas divisum. No significant difference was observed regarding patients' gender [5]. The higher prevalence of Santorinicele in elderly patients suggests it is likely acquired. However, Santroinicele was reported in younger patients, including children, indicating it can also be congenital [2,15].

The proposed pathophysiology is that the Santorinicele causes relative stenosis at the minor papilla, which in combination with the unfused dorsal and ventral ducts, i.e., pancreas divisum, results in elevated intraductal pressure, pancreatic secretions outflow obstruction, and subsequently recurrent attacks of pancreatitis [1,15].

Non-invasive imaging procedures, namely MRCP and secretin-enhanced magnetic resonance cholangiopancreatography (S-MRCP), are usually utilized in practice to evaluate the pancreatic duct. MRCP clearly delineates the ductal anatomy and shows any focal dilation in addition to its ability to detect other obstructive causes like a mass or a stone [5-7]. However, S-MRCP was developed specifically to assess the pancreas and the pancreatic duct anatomy [16]. In a study comparing MRCP to S-MRCP in patients with pancreatic divisum, Santorinicele was found in 8.6% of patients when using MRCP, with detection increasing to 33% following the utilization of S-MRCP [7]. However, the use of S-MRCP is limited because of its higher cost and longer examination time [16]. Endoscopic ultrasound (EUS) and ERCP are used as well [17]. EUS plays a role in the diagnosis of Santorinicele as a minimally invasive diagnostic modality if MRI is nondiagnostic [4]. ERCP, although it is traditionally considered the gold standard, is usually avoided as a first-line investigation due to its potential complications [6].

In our case, a multiplanner multisequential MRCP was performed, and heavily T2-weighted images with fat saturation were obtained with 3D reformations of the biliary and pancreatic ducts, in which Santorinicele and pancreas divisum were clearly demonstrated.

A CT scan of the upper abdomen in the portovenous phase can delineate the pancreatic duct and depict ductal anatomical variants to a limited extent. Even though CT scans are not accurate in diagnosing pancreatic ductal anomalies, assessing the pancreas anatomy on CT can be helpful, as it is commonly the initial imaging investigation for most patients with suspected pancreatitis [18,19].

Minor papilla sphincterotomy is a highly effective procedure for lowering the risk of pancreatitis in patients with Santorinicele [20]. To further minimize the likelihood of post-sphincterotomy pancreatitis, pancreatic stent placement is recommended following the procedure [4].

## Conclusions

Santorinicele with or without pancreas divisum is an uncommon cause of recurrent attacks of acute pancreatitis and should be looked for when investigating such patients, especially when no other cause can be identified. MRI of the upper abdomen with good technique and proper protocol can readily show the condition and any associated ductal variation or pathology.
